# Reliability of Health-Related Physical Fitness Tests among Colombian Children and Adolescents: The FUPRECOL Study

**DOI:** 10.1371/journal.pone.0140875

**Published:** 2015-10-16

**Authors:** Robinson Ramírez-Vélez, Diogo Rodrigues-Bezerra, Jorge Enrique Correa-Bautista, Mikel Izquierdo, Felipe Lobelo

**Affiliations:** 1 Centro de Estudios en Medición de la Actividad Física (CEMA), Escuela de Medicina y Ciencias de la Salud, Universidad del Rosario, Bogotá, D.C, Colombia; 2 Grupo GICAEDS, Facultad de Cultura Física, Deporte y Recreación, Universidad Santo Tomás, Bogotá, D.C, Colombia; 3 Public University of Navarra, Department of Health Sciences, Navarra, Pamplona, Spain; 4 Hubert Department of Global Health, Rollins School of Public Health, Emory University, Atlanta, Georgia, United States of America; University of Granada, SPAIN

## Abstract

Substantial evidence indicates that youth physical fitness levels are an important marker of lifestyle and cardio-metabolic health profiles and predict future risk of chronic diseases. The reliability physical fitness tests have not been explored in Latino-American youth population. This study’s aim was to examine the reliability of health-related physical fitness tests that were used in the Colombian health promotion “Fuprecol study”. Participants were 229 Colombian youth (boys n = 124 and girls n = 105) aged 9 to 17.9 years old. Five components of health-related physical fitness were measured: 1) morphological component: height, weight, body mass index (BMI), waist circumference, triceps skinfold, subscapular skinfold, and body fat (%) via impedance; 2) musculoskeletal component: handgrip and standing long jump test; 3) motor component: speed/agility test (4x10 m shuttle run); 4) flexibility component (hamstring and lumbar extensibility, sit-and-reach test); 5) cardiorespiratory component: 20-meter shuttle-run test (SRT) to estimate maximal oxygen consumption. The tests were performed two times, 1 week apart on the same day of the week, except for the SRT which was performed only once. Intra-observer technical errors of measurement (TEMs) and inter-rater (reliability) were assessed in the morphological component. Reliability for the Musculoskeletal, motor and cardiorespiratory fitness components was examined using Bland–Altman tests. For the morphological component, TEMs were small and reliability was greater than 95% of all cases. For the musculoskeletal, motor, flexibility and cardiorespiratory components, we found adequate reliability patterns in terms of systematic errors (bias) and random error (95% limits of agreement). When the fitness assessments were performed twice, the systematic error was nearly 0 for all tests, except for the sit and reach (mean difference: -1.03% [95% CI = -4.35% to -2.28%]. The results from this study indicate that the “Fuprecol study” health-related physical fitness battery, administered by physical education teachers, was reliable for measuring health-related components of fitness in children and adolescents aged 9–17.9 years old in a school setting in Colombia.

## Introduction

Physical fitness is a multi-dimensional construct that includes skill- and health-related components of which cardiorespiratory fitness (CRF) and muscular fitness, in particular, are powerful determinants of health in youth [1]. Substantial evidence indicates that children’s physical fitness levels are markers of their lifestyles and cardio -metabolic health profiles and also a predictor of the future risk of chronic diseases [2–6]. There are a number of cross-sectional studies and systematic reviews showing that low CFR young people is independently associated with a higher metabolic risk score [1,2,5]. Musculoskeletal component is inversely associated with metabolic risk [7,8] and is also a valuable indicator to monitor health in children [1] and adults [9]. Ruiz et al. [10] reported in a systematic reviews the relationship between neuromotor fitness (measures of muscular strength, flexibility, speed of movement and coordination) and health outcomes. Ortega et al. [1] indicated that lower-body power was inversely related to abdominal adiposity and that a composite strength score (with handgrip, standing broad jump, and an indicator of muscle endurance) was related to a positive lipid profile and improved glucose levels in female adolescents. For example, in children and adolescents neuromotor fitness was positively related to systolic blood pressure [β = 0.11; p<0.01] and inversely to the sum of four skinfolds [β = 0.21; p<0.01]. However, the authors also refer that they did not find evidence for other tests assessing motor fitness or flexibility due to the lack of studies [1,10].

Therefore, the inclusion of physical fitness in health surveillance systems is recommended by health authorities and schools may be an ideal setting for monitoring youth fitness [10]. Various test batteries are used to assess health-related fitness in young people globally, including the FITNESSGRAM battery [11], the President´s Challenge: Health Fitness [12], the EUROFIT [13], the CPAFLA [14], and the AFEA battery [15]. Recently, the Assessing Levels of Physical Activity health-related fitness test battery (ALPHA) was created as part of the framework for the European Union (EU)-funded HELENA Study (Healthy Lifestyle in Europe by Nutrition in Adolescence), to be used in public health monitoring in a comparable way within the EU [16]. The health-related fitness tests, included in the “Fuprecol study” (ASOCIACIÓN DE LA **FU**ERZA **PRE**NSIL CON MANIFESTACIONES TEMPRANAS DE RIESGO CARDIOVASCULAR EN NIÑOS Y ADOLESCENTES **COL**OMBIANOS. "ESTUDIO FUPRECOL" for Spanish), have been previously validated in youth [17–21]. However, the reliability of physical fitness tests has not been explored in Latino-American youth population. This is important to assess, particularly in the context of a low-to-middle income country (LMIC’s) setting like Colombia.

Surveillance of health-related fitness is particularly important in Latin America and other regions outside of high-income countries, where most of the burden due inactivity [22,23] and related non-communicable chronic diseases [24,25] occurs. Therefore, this study aimed to assess the reliability of health-related physical fitness field tests used in the “Fuprecol study” program among Colombian children and adolescents.

## Material and Methods

### Participants and Study Design

The present cross-sectional study was conducted to evaluate test-retest reliability, with 2 complete sets of assessments included in the “Fuprecol study”. Data were collected in a sample of students from one Colombian school (median age = 12.8 ±2.4 y, 46.2±12.4 kg, 1.50±0.1 m, 19.9±3.1 kg/m^2^), located in the Bogota capital district, in the Cundinamarca Department, Andean region. It is located at approximately 4°35′56″N 74°04′51″W, in an elevation of approximately 2,625 meters (min: 2,500, max: 3,250) above sea level. Bogota is considered an urban area, with approximately 7,862,277 habitants [26]. The contact for the raw data is available (S1 Appendix).

### Subjects

From a total sample of 270 youth who participated in the Fuprecol pilot study and performed all the physical fitness tests, a subsample of 229 healthy Colombian youth (boys n = 124 and girls n = 105), children and adolescents (9–17.9 years old) were asked to perform the tests again 3 days later. A sample of all individuals from the selected public school were invited to participate in the study. The participation rate was higher than 85%. A convenience sample of volunteers was included in groups by sex and age with 1-year increments (a total of 9 groups). Power calculations were based on the mean of the cardiorespiratory fitness of the first 30 participants in the ongoing data collection (range, 35–45 ml•kg•min^-1^) with a group SD of approximately 6.5 ml•kg•min^-1^. The significance level was set to 0.05, and the required power was set to at least 0.80. The sample size was estimated at 15 participants per age-sex group. The recruitment period lasted from June 2014 to January 2015.

The Review Committee for Research Human Subjects at the University of Rosario (Code N° CEI-ABN026-000262) approved the study. A comprehensive verbal description of the nature and purpose of the study and its experimental risks was given to the adolescents and their parents/guardians. All participants and their parents/legal guardians provided written informed consent before entering the study. The protocol was in accordance with the latest revision of the Declaration of Helsinki.

### Procedures

A manual of operations (study rationale, test procedures and how data were recorded) was designed for and read by the physical education teachers (*n = 24*) involved in the “Fuprecol study” before data collection started. Instructions provided to the participants were detailed in the manual of operations to standardize procedures. Participants were asked to perform each test two times. Tests were scheduled one week apart. Re-testing was performed at the same time of day to minimize circadian rhythm variability. The children and adolescents who took part in the re-test study did not differ in age, height, weight or body mass index (BMI) (P>0.05) from the adolescents who did not take part.

#### Morphological component

Weight was measured to the nearest 0.1 kg. Height was measured to the nearest 0.1 cm. Waist circumference (WC) was measured at the level of the umbilicus and the superior iliac crest. The measurement was made at the end of a normal expiration while the subject stood upright, with feet together and arms hanging freely at the sides. Hip circumference was measured over nonrestrictive underwear or light-weight shorts at the level of the maximum extension of the buttocks posteriorly in a horizontal plane, without compressing the skin. WC and hip circumferences were measured using a flexible steel tape (Lufkin Executive Thinline W 606). Instruments were calibrated to ensure acceptable accuracy. BMI was calculated as body weight in kilograms divided by the square of height in meters. Participants were categorized according to international BMI cut-off values as normal weight, overweight, and obese [27]. During the anthropometric measurements, students wore light clothing and were barefoot. Skinfold thicknesses were measured at the left side of the body to the nearest 0.1 mm using a Holtain skinfold caliper at the following sites: (1) triceps, halfway between the acromion process and the olecranon process, and (2) subscapular, approximately 20 mm below the tip of the scapula, at an angle of 45° to the lateral side of the body. For bioelectrical impedance analysis (BIA) measurements, a classical bipolar technique was used to estimate body fat (%) using a BIA-TANITA_®_ Model BF689 (Tanita, Tokyo, Japan) according to the manufacturer’s instructions.

#### Musculoskeletal component

**Explosive strength, Standing broad jump (cm):** The participant stood behind the starting line and was instructed to push off vigorously and jump as far as possible. The participant had to land with the feet together and stay upright. The test was repeated twice, and the best score was retained to the nearest 0.1 cm, as the distance between toes at take-off and heels at landing or whichever body part landed nearest to the take-off spot.

**Explosive strength, Vertical jump height (in cm):** The subjects stood with feet and toes on top of the measurement mat (Takei 5414 JUMP-DF DIGITAL VERTICAL^®^ Takei Scientific Instruments Co., Ltd, Niigata, Japan). The participants performed a countermovement jump. Two jumps were performed with one minute allowed for recovery between attempts.

**Handgrip strength (kg):** Handgrip strength was measured using a standard adjustable handle analogue handgrip dynamometer T-18 TKK SMEDLY III^®^ (Takei Scientific Instruments Co., Ltd, Niigata, Japan). Pupils were given a brief demonstration and verbal instructions for the test and if necessary the dynamometer was adjusted according to the child’s hand size according to predetermined protocols [16]. Handgrip strength was measured with the subject in a standing position with the shoulder adducted and neutrally rotated and arms parallel but not in contact with the body. The participants were asked to squeeze the handle for a maximum of 3–5 seconds, but no verbal encouragement was given during the test. Two trials were allowed in each limb and the average score recorded as peak grip strength (kg). Thus, the values of handgrip strength presented here combine the results of left- and right-handed subjects, without consideration of hand dominance.

#### Motor component

**Speed/agility test (speed of movement, agility and coordination assessment):** Two parallel lines were drawn on the floor 10 m apart. The adolescent ran as fast as possible from the starting line to the other line and returned to the starting line, crossing each line with both feet every time. This was performed twice, covering a distance of 40 m (4x10 m). Every time the adolescent crossed any of the lines, he/she should pick up (the first time) or exchange (second and third time) a sponge that had earlier been placed behind the lines. The stopwatch was stopped when the adolescent crossed the end line with one foot. The time taken to complete the test was recorded to the nearest tenth of a second. A slip-proof floor, four cones, a stopwatch and three sponges were used to perform the test.

#### Flexibility component

Hamstring and lumbar extensibility was measured using the sit and reach test. Participants were asked to sit on the floor with legs out straight ahead. Feet with shoes off were placed with the soles flat against the test device and shoulder-width apart. Both knees were held flat against the floor. With hands on top of each other and palms facing down, the patient reached forward along the measuring line as far as possible. The measuring stick on the device has the zero mark at 25 cm before the feet. The result was recorded directly from the meter on the device [13].

#### Cardiorespiratory component

**Cardiorespiratory fitness, 20-m shuttle run (ml•kg•min^-1^):** Participants ran in a straight line between two lines 20 m apart while keeping pace with pre-recorded audio signals. The initial speed was 8.5 km/hour and increased by 0.5 km/hour per minute. The test was finished when the participant failed to reach the end lines keeping pace with the audio signals on two consecutive occasions or when the subject stopped because of fatigue. The results were recorded to the nearest stage (minute) completed. To estimate VO_2_max using the 20-m shuttle run, we used the equation developed by Leger et al. [28] [VO_2max_ = 31.025 + 3.238 x V-3.248 x A + 0.1536 x V x A]. Here, “V” accounts for velocity (corresponding to that stage speed = 8 + 0.5 stage number, in km/h^-1^) of the last completed stage and “A” accounts for the subject’s age (in years).

The rationale for selecting these tests has been described elsewhere [16]. All tests were performed twice, and the best score was retained, except for the 20-m shuttle run test, which was performed only once. All standard operation procedures and protocols for fitness testing are available in the manual and videos in the ALPHA project website (https://sites.google.com/site/alphaprojectphysicalactivity/). With regard to the communication processes and explaining the tests, direct and simple oral instructions were used. When necessary, the evaluators provided visual models and examples before performing the tests. The participants did not previously receive specific training for these tests.

### Statistical analyses

The data are presented as the means±SD, unless otherwise stated. The morphological component was used as the indicator of precision by technical error of measurement (TEM). It is based on at least two measurements taken of the same child by the same observer (intra-observer variability) or by at least two observers taking the same measurement of the same child (inter-observer variability). The calculations for intra- and inter-observer error are broadly the same. The coefficient of reliability (R) estimates the proportion of between-subject variance in a measured population that is free from measurement error. Measures of R can be used to match the relative reliability of different anthropometric measurements, as well as of the same measurements in different age groups and to estimate sample size requirements in anthropometric studies. R as a percentage (R%) was calculated using the following equation: R% = 1-(total TEM^2^/SD^2^). To compare TEMs assessed for different measurements or different populations, absolute TEM was converted into relative TEM (%TEM) using the following equation: %TEM = (TEM/mean) x 100.

Musculoskeletal, motor and cardiorespiratory reliabilities were examined with potential systematic bias (H0; mean inter-trial difference = 0; H1; mean inter-trial difference≠0). Sex differences of the studied physical fitness tests were analyzed by a one-way analysis of variance (ANOVA) on inter-trial difference (test 2-test 1, hereafter called T2-T1) with sex as a fixed factor. As no sex-specific effect on reliability of the studied physical fitness tests was found, the analyses were performed for both boys and girls together. Reliability was also assessed according to the method of Bland-Altman. The analysis measures bias as estimated from mean differences, the 95% confidence interval for bias, the limits of agreement and ± 1.96 SD. of the difference. All calculations were performed using SPSS v.15.0 software for Windows (SPSS, Chicago, Illinois, USA). For all analyses, the significance level was 0.05.

## Results

The characteristics for the four components of the “Fuprecol study” (mean values±SD) assessed twice, as well as the mean inter-trial difference in the studied boys and girls, children and adolescents, are shown in Table 1. Neither systematic bias nor sex differences were found for any of the studied tests.

**Table 1:** Reliability of “Fuprecol study” of morphologic, musculoskeletal, motor and cardiorespiratory component (mean±SD) in boys (n = 124) and girls (n = 105) from Bogota, Colombia.

Component								
	1st Trial (T1)		2nd Trial (T2)		Inter-trial difference (T2_T1)			
	Boys	Girls	Boys	Girls	Boys	P value Boys	Girls	P value Girls
**Morphologic**								
Age (years)	12.8 ± 2.4	12.8 ± 2.5	-	-	-		-	
Weight (kg)	47.1 ± 13.6	45.3 ± 11.1	-	-	-		-	
Height (m)	1.53 ± 0.2	1.49 ± 0.1	-	-	-		-	
Body mass index (kg/m^2^)	19.8 ± 3.1	20.1 ± 3.3	-	-	-		-	
Waist circumference (cm)	65.4 ± 7.8	63.5 ± 7.4	-	-	-		-	
Hip circumference (cm)	81.2 ± 9.1	82.5 ± 10.3	-	-	-		-	
Triceps skinfolds (mm)	17.0 ± 5.4	21.0 ± 5.8	-	-	-		-	
Subscapular skinfolds (mm)	15.5 ± 5.7	18.1 ± 5.9	-	-	-		-	
Body fat by BIA (%)	16.5 ± 9.1	26.0 ± 21.4	-	-	-		-	
**Musculoskeletal**								
Handgrip (kg)^a^	19.6 ± 8.9	16.9 ± 5.1	19.0 ± 8.5	16.5 ± 5.4	0.6 ± 2.0	0.209	0.4 ± 1.6	0.059
Standing broad jump (cm)	143.8 ± 31.5	111.7 ± 21.5	140.3 ± 32.7	110.5 ± 18.1	3.5 ± 18.1	0.053	1.1 ± 15.3	0.065
Vertical jump (cm)	32.6 ± 14.6	24.9 ± 6.7	33.0 ± 8.0	27.0 ± 4.5	-0.4 ± 12.9	0.761	-2.1 ± 5.6	0.056
**Motor**								
Sit and reach (cm)	21.1 ± 6.7	24.7 ± 7.8	20.2 ± 6.1	24.2 ± 7.8	0.9 ± 4.2	0.056	0.4 ± 4.7	0.064
4x10m shuttle run (s)	13.4 ± 1.6	14.9 ± 1.6	13.7 ± 1.6	15.1 ± 1.3	-0.3 ± 1.5	0.094	-0.2 ± 1.8	0.365
**Cardiorespiratory**								
20-m shuttle run (stage)	3.9 ± 2.3	2.2 ± 1.8	4.1 ± 2.3	2.4 ± 1.2	-0.1 ± 0.8	0.079	-0.2 ± 0.7	0.067

^a^ The average of right and left side scores is shown in the table and was used for the analyses. BIA: bioelectrical impedance analysis

Table 2 shows the inter-observer TEM and %R for each morphologic component variable. The TEMs were 0.51 kg for the weight, 0.01 cm for the height, 0.34 kg/m^2^ for the BMI, 0.86 cm for the WC, 0.91 cm for the hip circumference, and 0.63% for the body fat by BIA and ranged between 0.59 and 0.60 mm for the skinfolds measured (triceps and subscapular, respectively). Reliability for anthropometric measurements were always higher than 95%.

**Table 2:** Inter-observer TEM, relative TEM and intra-observer morphologic component assessments of children and adolescents from Bogota, Colombia.

	Mean	Inter-observer		Intra-observer
		TEM	%TEM	R%
Weight (kg)	45.9	0.5104	1.1101	0.9615
Height (m)	1.51	0.0192	1.2716	0.9965
Body mass index (kg/m^2^)	19.4	0.3401	1.7446	0.9854
Waist circumference (cm)	65.3	0.8655	1.3240	0.9795
Hip circumference (cm)	82.5	0.9105	1.1030	0.9818
Triceps skinfolds (mm)	18.4	0.5984	3.2488	0.9764
Subscapular skinfolds (mm)	15.8	0.6070	3.8391	0.9799
Body fat by BIA (%)	19.6	0.6392	3.2526	0.9856

TEM: technical error of measurement; BIA: bioelectrical impedance analysis

**Fig 1:**
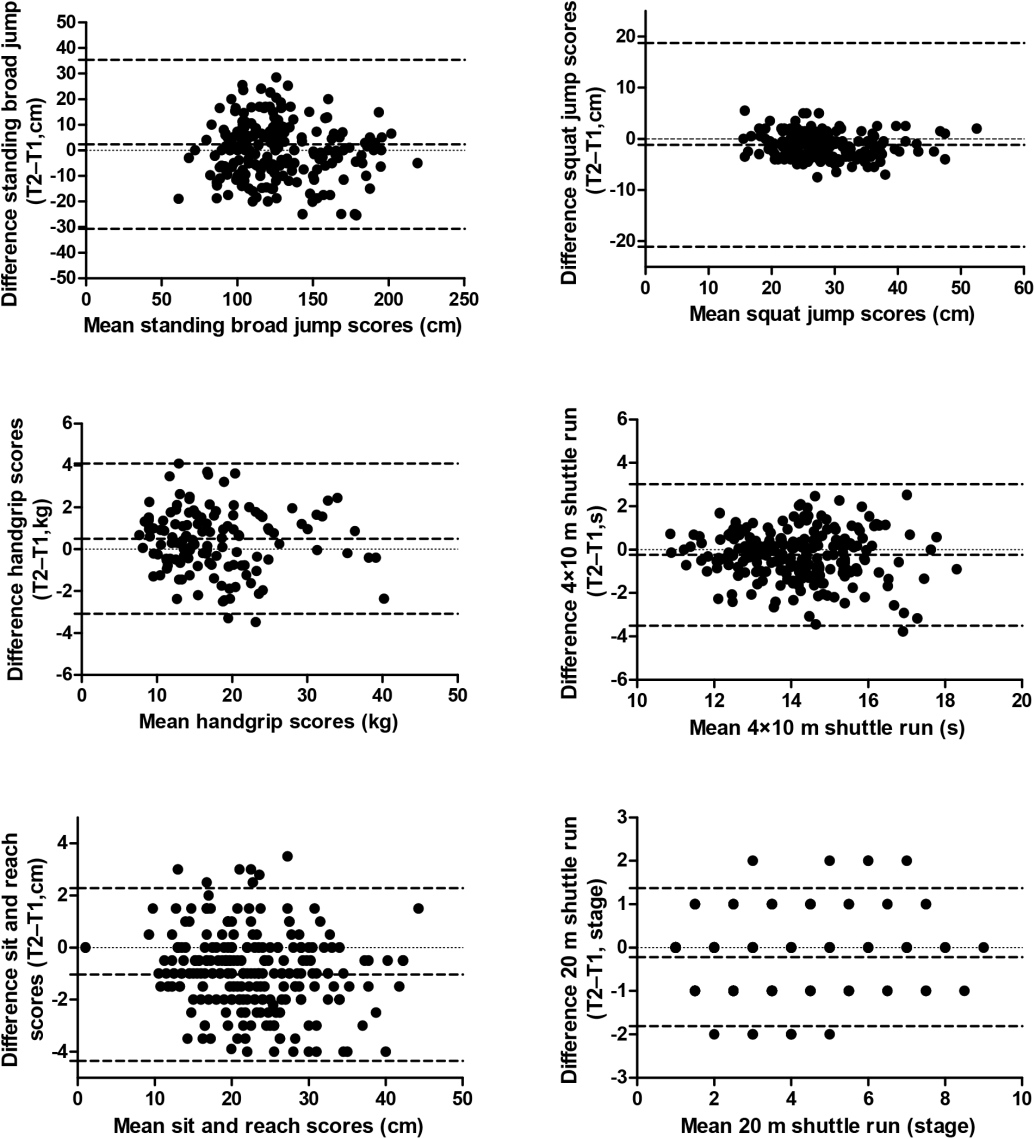
Bland–Altman plot of the handgrip, standing broad jump, vertical jump, sit and reach, 4x10m shuttle run, 20-m shuttle run of children and adolescents from Bogota, Colombia. The central dotted line represents the mean differences between the second trial (T2) and the first trial (T1); the upper and lower dotted lines represent the upper and lower 95% limits of agreement (mean differences ± 1.96 SD of the differences), respectively.

The Bland–Altman plots (Fig 1) graphically show the reliability patterns, in terms of systematic errors (bias or mean inter-trial differences) and random error (95% limits of agreement), of the physical fitness tests studied. The systematic error when fitness assessment were performed twice was nearly 0 for all the tests, except for the sit and reach (mean difference: -1.03% [95%CI = -4.35% to 2.28%]).

## Discussion

Our study’s main finding shows that the “Fuprecol study” of health-related fitness battery administered by physical education teachers is reliable for assessing the levels of physical fitness in youth in a school environment in the Colombian setting. Measurement of the morphological component through anthropometry is less expensive and more practical than other techniques, and thus, it seems to be the most adequate method for epidemiological studies with a high number of subjects. Therefore, it is extremely important to define the reliability of body fat and fat distribution methods [29]. However, to our knowledge, this is the first study assessing the reliability of health-related fitness tests, including its five components (morphological, musculoskeletal motor, flexibility and cardiorespiratory) in Latin American youth.

Validity refers to the ability of the test to reflect what it is designed to measure [17]. The validity of fitness test batteries is judged by comparing to the “gold standard” method [18]. Reliability refers to the reproducibility of values of a test in repeated trials on the same individual [19]. In the “Fuprecol study”, both the intra-rater and inter-rater TEM and %R values were above the required levels. Specifically, the TEMs for height and the waist and hip circumferences were frequently lower than 1 cm and the %R greater than 95% (intra-rater), whereas the inter-rater TEM and %R were more susceptible to error. Our results are similar to those found in other studies carried out with European adolescents “The HELENA study” [30], Spanish adolescents (Sedentary behaviors and socio-economic status in Spanish adolescents **-**AVENA study**-**) [31], children from eight European countries (Identification and prevention of Dietary and lifestyle-induced health Effects In Children and Infants- IDEFICS study-) [32] and a recent European multi-country study to develop an obesity prevention program specifically for pre-school children (ToyBox study) [33]. In the ALPHA project framework, Artero et al. [34] noted that, in general, (a) the reliability was higher for BMI and body circumference compared with skinfolds, (b) the variability of skinfold measurements is greater when several measures are taken, and (c) the knowledge of protocols and years of experience could significantly predict precision and accuracy in measuring skinfold thicknesses. Allowance for measurement error might be up to 10% of the observed variance equivalent to an R value of 90% or greater [35]. Although this might be an acceptable lower limit, even at R values of approximately 95%, there is the occasional gross error. Only when R is 99% is such an error unlikely [36]. In addition, these studies reported significant correlations between the means and SD of skinfold thickness and circumferences, which indicates that the variability of these measurements could be greater when there are several measures taken (heteroscedasticity) [17,30,31].

On the other hand, for the musculoskeletal, motor and cardiorespiratory components, we found adequate reliability patterns, in terms of systematic errors (bias) and random error (95% limits of agreement). Our findings are in agreement with previous results from Romero-España et al. [20] and Ortega et al. [17], who examined the reliability of health-related fitness tests (handgrip, vertical jump and standing long jump) among children (age range: 6–11.9 years) and adolescents (age range: 12–18 years), respectively. In general, evidence indicates that strength test produces have moderate test-retest reliability. For example, when performing handgrip strength test with the TKK dynamometer adapted to the hand size, the agreement between test and retest is the same throughout the range of measured values (homoscedasticity) [17,34]. In addition, evidence has shown no significant differences on a test-retest for the SBJ (−0.3 ± 12.9 cm for boys and 0.3 ± 9 cm for girls), for boys and girls aged on average 13.6 years old [17]. Ortega et al. [17] and Artero et al. [34] added that neither learning nor fatigue effects were found and that the SBJ test is reliable for the assessment of lower body muscular strength for both male and female adolescents. On the other hand, the vertical jump is a movement where an athlete jumps vertically to achieve the highest point above the ground with 3 studies including it in the assessment battery of healthy children and adolescents [37–39]. Over the years, literature has validated this test confirming both feasibility, reliability and accuracy, even though the “Abalakov jump” is more specific for sports in which maximal strength is expressed on a vertical plane [40,41]. In our study was used the Takei 5414 JUMP-DF DIGITAL VERTICAL^®^ measurement platform and we found a systematic error of -0.217% (95% limits of agreement = -1.808% to 1.357%). Previous studies have analyzed the reliability in countermovement vertical jumps. Moir et al. [42] analyzed the vertical jump height in women and men using a contact mat with sessions across a 4-week period, with each session separated by 1 week, showing adequate reliability (ICC = 0.87 to 0.93). Finally, Nuzzo et al. [43] analyzed the reliability of the Just Jump System, and Myotest to measure countermovement vertical jump (CMJ) height. The tests showed an excellent intrasession reliability (ICC = 0.95; SEM = 1.5 cm) and good intersession reliability (ICC = 0.88; SEM = 2.4 cm; limits of agreement = -0.08 ± 4.06 cm).

The 20 mSRT has been initially validated by Léger and Gadoury [28], who found a correlation coefficient of (r = 0.84) against a standard laboratory maximal oxygen consumption (VO_2max_). The reliability of tests assessing cardiorespiratory fitness has been investigated in previous studies [17,34,44–46]. The systematic error for the 20 mSRT was nearly 0. There was strong evidence indicating that the 20 mSRT produces results with good test-retest reliability (ICC range 0.78 to 0.93) in children and adolescents aged 8–18 years [17,34].

Lastly, our results based on the heteroscedasticity analysis and Bland-Altman plots indicate that the worse the performance in the sit and reach tests, the worse the degree of the agreement (mean difference: -1.03% [95%CI = -4.35% to 2.28%]). The reliability of the sit and reach test was analyzed in previous studies [17,47]. Contrary to our findings, Hartman [48] demonstrated good test-retest reliability (ICC = 0.96 to 0.99) in 87 boys and 92 girls aged 6–12 years. However, they also indicated that those adolescents who scored lower presented a significantly higher (P < 0.05) test-retest difference compared with those who scored higher (i. e., heteroscedasticity) [48]

Latin-American populations have disparities in health along with disparities in modifiable risk factors, including low participation in physical activity. For instance, it has been said that inequalities in nutrition could be related to race/ethnicity inequalities and household characteristics [49]. While the relationship between race/ethnicity and obesity in children is inconsistent in high-income countries, in LMIC’s there appears to be a strong positive association between the two [49]. The obesity-race/ethnicity association could vary by sex, age, and country [48]. LMICs, such as Colombia, are experiencing rapid urbanization and integration to global markets, which lead to changes in diet and physical activity and, with these changes, large effects on body composition and other health-related fitness components [49–52]. These changes are contributing to a global increase in the prevalence of noncommunicable diseases [49]. Therefore, the inclusion of fitness within health surveillance systems is justifiable and has been recommended [53].

In conclusion, the “Fuprecol study” was conducted with standardized methodology among Colombian children and adolescents. Intra- and inter-observer reliabilities of the morphologic component of fitness surpassed 95%. In addition, we found adequate reliability patterns for musculoskeletal, motor and cardiorespiratory components, in terms of systematic errors (bias) and random error (95% limits of agreement). It is necessary to highlight that all these tests have shown to be reliable, yet the evidence for some of them is limited due to the low number of studies, in particular in LMICs. Future multicenter studies in Latin America should ensure that standardization and training procedures are in place.

## Supporting Information

S1 Appendix Data availability statement. (DOCX)
