# Supplementary material for: Reliability of Health-Related Physical Fitness Tests among Colombian Children and Adolescents: The FUPRECOL Study
Source: PLoS One. 2015 Oct 16;10(10):e0140875. doi: 10.1371/journal.pone.0140875 (PMC4608730; doi:10.1371/journal.pone.0140875)
Supplement: S1 Appendix — (DOCX) [file pone.0140875.s001.docx]

### S1 Appendix. Data availability statement

All relevant data are within the paper.

 "The study data have legal restrictions and ethical imposed by the authors' IRB (UNIVERSIDAD DEL ROSARIO).

The explanation of these restrictions is because they are children and adolescent students of Public Schools Colombia (Law Nº 1.581, Octuber 2012 and National Decret Nº 1377 de 2013). Avalaible: <http://www.alcaldiabogota.gov.co/sisjur/normas/Norma1.jsp?i=49981> and <http://www.oei.es/quipu/colombia/codigo_infancia.pdf>

Contact for the raw data.

| **Study** | **Details of data access / requests** |
| --- | --- |
| Universidad Santo Tomas (USTA) | The initial contact point for collaborations is Professor Robinson Ramirez-Velez (robinsonramirez@usantotomas.edu.co) |

References (for data access arrangements)

1. GICAEDS Group. Physical Activity and Health Research Branch. Allied Instituto Colombiano para el Desarrollo de la Ciencia y la Tecnología “Francisco José de Caldas” COLCIENCIAS, 2014-2015 and Centro de Estudios en Medición de la Actividad Física (CEMA), Universidad del Rosario [computer file]. Bogota, Colombia: USTA Data Archive [distributor], May 2015. “Fuprecol study” (ASOCIACIÓN DE LA **FU**ERZA **PRE**NSIL CON MANIFESTACIONES TEMPRANAS DE RIESGO CARDIOVASCULAR EN NIÑOS Y ADOLESCENTES **COL**OMBIANOS. "ESTUDIO FUPRECOL" for Spanish).
